# (8-Benzoyl-2,7-dimeth­oxy­naphthalen-1-yl)(4-phen­oxy­phen­yl)methanone

**DOI:** 10.1107/S1600536813004303

**Published:** 2013-02-20

**Authors:** Kosuke Sasagawa, Rei Sakamoto, Ryo Takeuchi, Noriyuki Yonezawa, Akiko Okamoto

**Affiliations:** a Department of Organic and Polymer Materials Chemistry, Tokyo University of Agriculture & Technology, Koganei, Tokyo 184-8588, Japan

## Abstract

In the mol­ecule of the title compound, C_32_H_24_O_5_, the benzoyl group and the 4-phenoxy substituted benzoyl group at the 1- and 8-positions of the naphthalene ring system are aligned almost anti­parallel. The two benzene rings make a dihedral angle of 21.18 (10)°, and are inclined to the naphthalene ring system by 86.53 (9) and 82.95 (8)°, respectively. In the crystal, C—H⋯O inter­actions are observed involving aromatic and meth­oxy H atoms with ketonic carbonyl O atoms, as well as C—H⋯π inter­actions between aromatic H atoms and the π-systems of naphthalene and benzene rings. These interactions form a three-dimensional architecture and afford a waved alignment of the naphthalene ring systems along the *c* axis.

## Related literature

For the synthesis of aroylated naphthalene compounds *via* electrophilic aromatic substitution of naphthalene derivatives, see: Okamoto & Yonezawa (2009 ▶); Okamoto *et al.* (2011 ▶). For the structures of closely related compounds, see: Nakaema *et al.* (2008 ▶); Hijikata *et al.* (2010 ▶); Sasagawa *et al.* (2011 ▶, 2013 ▶); Muto *et al.* (2012 ▶).
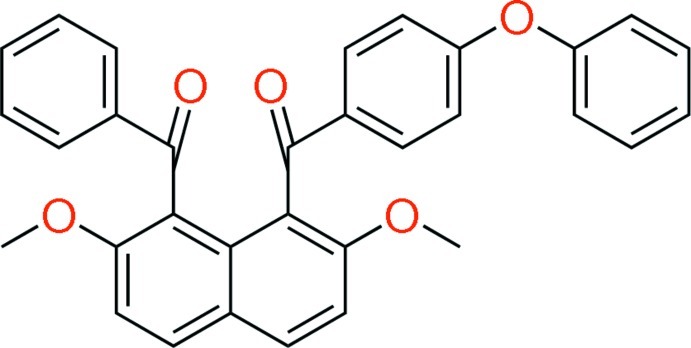


## Experimental

### 

#### Crystal data

C_32_H_24_O_5_
*M* *_r_* = 488.51
Orthorhombic, 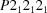

*a* = 8.19645 (10) Å
*b* = 11.5051 (2) Å
*c* = 26.4916 (4) Å
*V* = 2498.18 (7) Å^3^
*Z* = 4
Cu *K*α radiation
μ = 0.71 mm^−1^
*T* = 193 K
0.60 × 0.30 × 0.20 mm

#### Data collection

Rigaku R-AXIS RAPID diffractometer
Absorption correction: multi-scan (*ABSCOR*; Rigaku, 1995 ▶) *T*_min_ = 0.802, *T*_max_ = 0.868
46867 measured reflections
2605 independent reflections
2543 reflections with *I* > 2σ(*I*)
*R*_int_ = 0.030

#### Refinement

*R*[*F* ^2^ > 2σ(*F* ^2^)] = 0.032
*wR*(*F* ^2^) = 0.085
*S* = 1.04
2605 reflections
337 parameters
H-atom parameters constrained
Δρ_max_ = 0.19 e Å^−3^
Δρ_min_ = −0.14 e Å^−3^

### 

Data collection: *PROCESS-AUTO* (Rigaku, 1998 ▶); cell refinement: *PROCESS-AUTO*; data reduction: *PROCESS-AUTO*; program(s) used to solve structure: *Il Milione* (Burla *et al.*, 2007 ▶); program(s) used to refine structure: *SHELXL97* (Sheldrick, 2008 ▶); molecular graphics: *ORTEPIII* (Burnett & Johnson, 1996 ▶); software used to prepare material for publication: *SHELXL97*.

## Supplementary Material

Crystal structure: contains datablock(s) I, global. DOI: 10.1107/S1600536813004303/vm2188sup1.cif

Structure factors: contains datablock(s) I. DOI: 10.1107/S1600536813004303/vm2188Isup2.hkl

Supplementary material file. DOI: 10.1107/S1600536813004303/vm2188Isup3.cml

Additional supplementary materials:  crystallographic information; 3D view; checkCIF report

## Figures and Tables

**Table 1:** Hydrogen-bond geometry (Å, °) *Cg*1, *Cg*2 and *Cg*3 are the centroids of the C27–C32, C12–C17 and C5–C10 rings, respectively.

*D*—H⋯*A*	*D*—H	H⋯*A*	*D*⋯*A*	*D*—H⋯*A*
C30—H30⋯O5^i^	0.95	2.52	3.423 (3)	158
C7—H7⋯O5^ii^	0.95	2.37	3.304 (3)	168
C25—H25*B*⋯O5^ii^	0.98	2.34	3.122 (3)	136
C3—H3⋯O1^iii^	0.95	2.40	3.310 (2)	160
C20—H20⋯*Cg*1^iv^	0.95	2.84	3.652 (3)	144
C23—H23⋯*Cg*2^v^	0.95	2.76	3.628 (2)	151
C29—H29⋯*Cg*3^i^	0.95	2.85	3.652 (3)	142

Symmetry codes: (i) 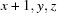; (ii) 
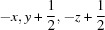
; (iii) 
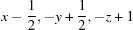
; (iv) 
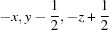
; (v) 
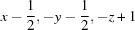
.

**Fig. 1.**
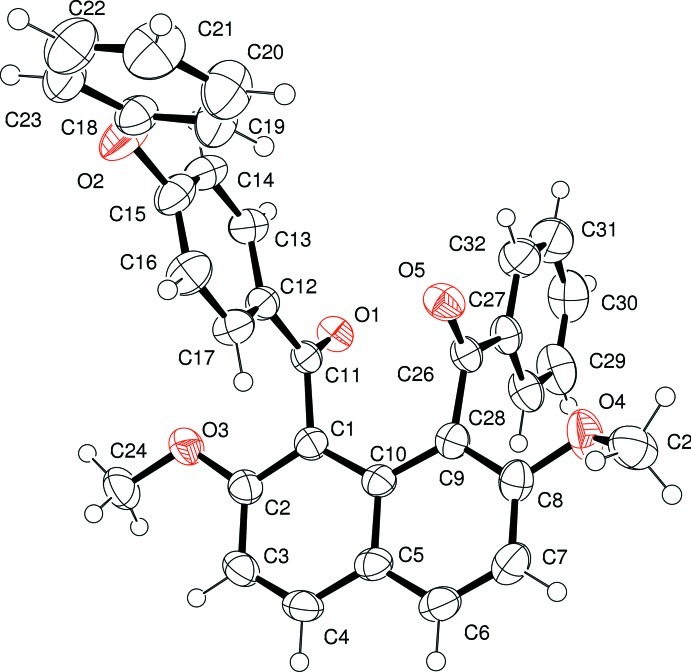
Molecular structure with the atom-labeling scheme and displacement ellipsoids drawn at the 50% probability level. Hydrogen atoms are shown as small spheres of arbitrary radius.

**Fig. 2.**
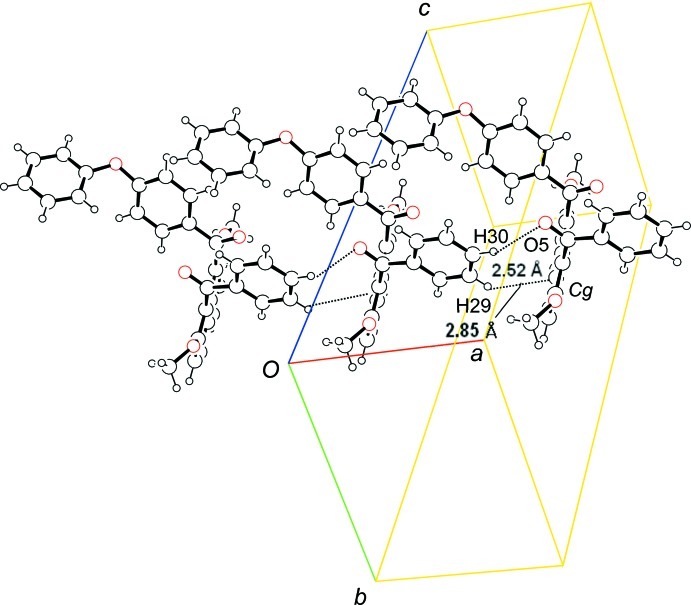
Intermolecular C—H···O interactions between H30 and O5 [symmetry code: 1 + x, y, z] along the a axis (dashed lines).

**Fig. 3.**
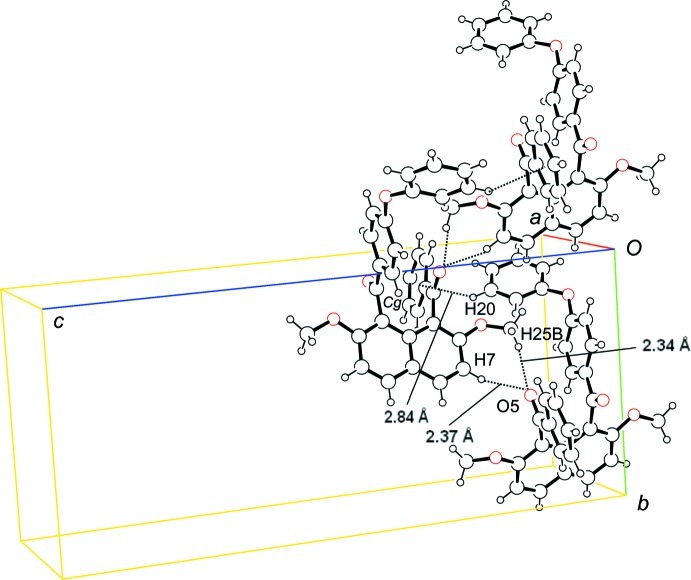
Intermolecular C—H···O interactions between H25B and O5, H7 and O5 [symmetry code: -x, 1/2 + y, 1/2 - z] along the b axis (dashed lines).

**Fig. 4.**
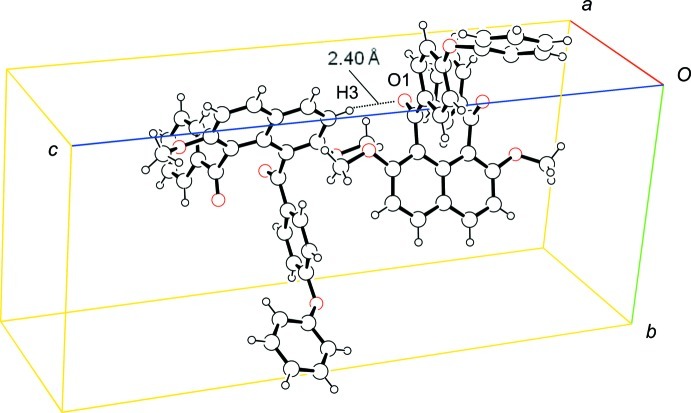
Intermolecular C—H···O interactions between H3 and O1 [symmetry code: -1/2 + x, 1/2 - y, 1 - z] along the c axis (dashed lines).

## supplementary crystallographic information

### Comment

In the course of our study on selective electrophilic aromatic aroylation of the naphthalene ring core, 1,8-diaroylnaphthalene compounds have proved to be formed regioselectively by the aid of a suitable acidic mediator (Okamoto & Yonezawa, 2009, Okamoto *et al.*, 2011). Recently, we have reported the X-ray crystal structures of 1,8-diaroylated 2,7-dimethoxynaphthalene derivatives such as 1,8-dibenzoyl-2,7-dimethoxynaphthalene (Nakaema *et al*., 2008), {8-[4-(butoxy)benzoyl]-2,7-dimethoxynaphthalen-1-yl}[4-(butoxy)phenyl] methanone [1,8-bis(4-butoxylbenzoyl)-2,7-dimethoxynaphthalene] (Sasagawa *et al.*, 2011) and [2,7-dimethoxy-8-(4-methoxybenzoyl)-naphthalen-1-yl](4-methoxyphenyl) methanone chloroform monosolvate [1,8-bis(4-methoxybenzoyl)-2,7-dimethoxynaphthalene] (Sasagawa *et al.*, 2013).

The aroyl groups in the 1,8-diaroylnaphthalene compounds are almost perpendicular to the naphthalene rings, and oriented in opposite directions (*anti*-orientation). According to our knowledge, most 1,8-diaroylnaphthalene derivatives have *anti*-oriented structures. Recently, we have also clarified another structure of the 1,8-diaroylnaphthalene derivatives, in which the two aroyl groups are situated in same direction (*syn*-orientation), [2,7-dimethoxy-1,8-bis(4-phenoxybenzoyl)naphthalene; Hijikata *et al.*, 2010].

Moreover, we have reported the asymmetric 1,8-diaroylnaphthalene derivatives, [8-(4-chlorobenzoyl)-2,7-dimethoxynaphthalen-1-yl](2,4,6-trimethylphenyl) methanone (Muto *et al.*, 2012). As a part of our ongoing studies on the molecular structures of these kinds of asymmetric homologous molecules, the X-ray crystal structure of the title compound, 2,7-dimethoxynaphthalene bearing benzoyl and phenoxybenzoyl groups at the 1,8-positions, is discussed in this article.

The molecular structure of the title compound is displayed in Fig 1. The benzoyl group and 4-phenoxybenzoyl group are situated in the *anti*-orientation. The dihedral angle between the best planes of the benzene rings of two kinds of benzoyl groups is 21.18 (10) °. The dihedral angles of the best planes of the benzene rings of the benzoyl moiety and 4-phenoxybenzoyl moiety with the naphthalene ring are 86.53 (9) and 82.95 (8) °, respectively. In addition, the dihedral angle between both benzene rings of the 4-phenoxyphenyl moiety is 69.19 (10) °.

The two ketonic carbonyl moieties (C11=O1, C26=O5) and the benzene ring of two benzoyl moieties lie in the same plane [torsion angle O1—C11—C12—C13 = 7.3 (2) °, O5—C26—C27—C32 = 1.3 (3) °]. In the crystal, C—H···O and C—H···π interactions effectively contribute to the stabilization of the molecular packing (Table 1): a C—H···O interaction between the hydrogen atom of the 4-position of the benzoyl group and the oxygen atom of the carbonyl moiety in the benzoyl group, a C—H···π interaction between the hydrogen atom of the 3-position of the benzoyl group and the π-system of the naphthalene ring (Fig. 2), C—H···O interactions of a hydrogen atom of the methoxy group at the 2-position of the naphthalene ring and a hydrogen atom at the 3-position of the naphthalene ring with the carbonyl oxygen atom of the benzoyl group, a C—H···π interaction between the hydrogen atom of the 3-position of the phenoxy moiety and the π-system of the benzoyl group (Fig. 3), a C—H···O interaction between the hydrogen atom at the 6-position of the naphthalene ring and the carbonyl oxygen atom of the phenoxybenzoyl group (Fig. 4), and a C—H···π interaction between the hydrogen atom of the 6-position of the phenoxy moiety and the π-system of the internal benzene ring of 4-phenoxybenzoyl group.

### Experimental

In a 10 ml one-necked flask, [8-(4-benzoyl)-2,7-dimethoxynaphthalen-1-yl](4-fluorophenyl)-methanone (1.0 mmol, 414 mg), phenol (1.5 mmol, 141 mg), potassium carbonate (2.5 mmol, 346 mg) and freshly distilled dimethylacetamide (2.5 ml) were stirred at 423 K for 6 h. This mixture was poured into 2*M* aqueous HCl (100 ml). The aqueous layer was extracted with ethyl acetate (20 ml × 3). The combined extracts were washed with water followed by washing with brine. The extracts thus obtained were dried over anhydrous MgSO_4_. The solvent was removed under reduced pressure to give a cake (yield 88%). The crude product was purified by recrystallization from methanol (yield 47%). Colorless platelet single crystals suitable for X-ray diffraction were obtained by repeated crystallization from ethanol.

Spectroscopic Data:

^1^H NMR *δ* (500 MHz, CDCl_3_): 3.69 (3*H*, s), 3.72 (3*H*, s), 6.86 (2*H*, d, *J* = 8.5 Hz), 7.09 (2*H*, d, *J* = 7.5 Hz), 7.16–7.22 (3*H*, m), 7.32–7.71 (4*H*, m), 7.50 (1*H*, t, *J* = 7.5 Hz), 7.64–7.71 (4*H*, m), 7.94 (1*H*, d, *J* = 9.0 Hz), 7.95 (1*H*, d, *J* = 9.0 Hz) p.p.m.

^13^C NMR δ (125 MHz, CDCl_3_): 56.39, 56.45, 111.18, 116.70, 120.28, 121.43, 121.46, 124.33, 124.34, 125.49, 127.94, 129.07, 129.66, 129.86, 131.38, 131.93, 131.99, 132.57, 133.49, 138.59, 155.52, 156.07, 156.23, 161.51, 195.29, 196.72 p.p.m.

IR (KBr): 1659 (C=O), 1599, 1580, 1512 (Ar), 1240 (OMe) cm^-1^

HRMS (m/*z*): [*M*+H]^+^ calcd. for C_32_H_25_O_5,_ 489.1702, found, 489.1690

m.p. = 466.1—469.7 K

### Refinement

All H atoms were found in a difference map and were subsequently refined as riding atoms, with C—H = 0.95 (aromatic) and 0.98 (methyl) Å with *U*_ĩso_(H) = 1.2 *U*_eq_(C).

### Crystal data

**Table:**

C_32_H_24_O_5_	*F*(000) = 1024
*M**_r_* = 488.51	*D*_x_ = 1.299 Mg m^−^^3^
Orthorhombic, *P*2_1_2_1_2_1_	Cu *K*α radiation, λ = 1.54187 Å
Hall symbol: P 2ac 2ab	Cell parameters from 44479 reflections
*a* = 8.19645 (10) Å	θ = 3.3–68.3°
*b* = 11.5051 (2) Å	µ = 0.71 mm^−^^1^
*c* = 26.4916 (4) Å	*T* = 193 K
*V* = 2498.18 (7) Å^3^	Block, colourless
*Z* = 4	0.60 × 0.30 × 0.20 mm

### Data collection

**Table:**

Rigaku R-AXIS RAPID diffractometer	2605 independent reflections
Radiation source: rotating anode	2543 reflections with *I* > 2σ(*I*)
Graphite monochromator	*R*_int_ = 0.030
Detector resolution: 10.000 pixels mm^-1^	θ_max_ = 68.3°, θ_min_ = 3.3°
ω scans	*h* = −9→9
Absorption correction: multi-scan (*ABSCOR*; Rigaku, 1995)	*k* = −13→13
*T*_min_ = 0.802, *T*_max_ = 0.868	*l* = −31→31
46867 measured reflections	

### Refinement

**Table:**

Refinement on *F*^2^	Secondary atom site location: difference Fourier map
Least-squares matrix: full	Hydrogen site location: inferred from neighbouring sites
*R*[*F*^2^ > 2σ(*F*^2^)] = 0.032	H-atom parameters constrained
*wR*(*F*^2^) = 0.085	*w* = 1/[σ^2^(*F*_o_^2^) + (0.0545*P*)^2^ + 0.3758*P*] where *P* = (*F*_o_^2^ + 2*F*_c_^2^)/3
*S* = 1.04	(Δ/σ)_max_ < 0.001
2605 reflections	Δρ_max_ = 0.19 e Å^−^^3^
337 parameters	Δρ_min_ = −0.14 e Å^−^^3^
0 restraints	Extinction correction: *SHELXL97* (Sheldrick, 2008), Fc^*^=kFc[1+0.001xFc^2^λ^3^/sin(2θ)]^-1/4^
Primary atom site location: structure-invariant direct methods	Extinction coefficient: 0.0019 (2)

### Special details

**Table:**

Geometry. All e.s.d.'s (except the e.s.d. in the dihedral angle between two l.s. planes) are estimated using the full covariance matrix. The cell e.s.d.'s are taken into account individually in the estimation of e.s.d.'s in distances, angles and torsion angles; correlations between e.s.d.'s in cell parameters are only used when they are defined by crystal symmetry. An approximate (isotropic) treatment of cell e.s.d.'s is used for estimating e.s.d.'s involving l.s. planes.
Refinement. Refinement of *F*^2^ against ALL reflections. The weighted *R*-factor *wR* and goodness of fit *S* are based on *F*^2^, conventional *R*-factors *R* are based on *F*, with *F* set to zero for negative *F*^2^. The threshold expression of *F*^2^ > σ(*F*^2^) is used only for calculating *R*-factors(gt) *etc*. and is not relevant to the choice of reflections for refinement. *R*-factors based on *F*^2^ are statistically about twice as large as those based on *F*, and *R*- factors based on ALL data will be even larger.

### Fractional atomic coordinates and isotropic or equivalent isotropic displacement parameters (Å^2^)

**Table:**

	*x*	*y*	*z*	*U*_iso_*/*U*_eq_	
O1	0.31166 (15)	0.05570 (11)	0.41889 (5)	0.0395 (3)	
O2	−0.2584 (2)	−0.31098 (13)	0.44425 (6)	0.0630 (5)	
O3	0.08516 (19)	0.16347 (12)	0.50987 (4)	0.0461 (3)	
O4	0.1135 (2)	0.25452 (15)	0.24234 (5)	0.0582 (4)	
O5	0.11094 (17)	0.02780 (12)	0.31322 (5)	0.0429 (3)	
C1	0.0961 (2)	0.19406 (14)	0.42338 (6)	0.0334 (4)	
C2	0.0552 (2)	0.23680 (15)	0.47042 (7)	0.0374 (4)	
C3	−0.0139 (3)	0.34833 (17)	0.47642 (7)	0.0429 (4)	
H3	−0.0370	0.3778	0.5091	0.051*	
C4	−0.0469 (2)	0.41295 (15)	0.43472 (7)	0.0432 (4)	
H4	−0.0976	0.4867	0.4387	0.052*	
C5	−0.0081 (2)	0.37364 (15)	0.38559 (7)	0.0381 (4)	
C6	−0.0466 (3)	0.44090 (16)	0.34258 (8)	0.0450 (4)	
H6	−0.0998	0.5136	0.3469	0.054*	
C7	−0.0093 (3)	0.40417 (18)	0.29486 (8)	0.0475 (5)	
H7	−0.0373	0.4501	0.2663	0.057*	
C8	0.0710 (2)	0.29759 (18)	0.28879 (7)	0.0416 (4)	
C9	0.1126 (2)	0.22877 (15)	0.32944 (6)	0.0347 (4)	
C10	0.0701 (2)	0.26357 (14)	0.37942 (6)	0.0328 (4)	
C11	0.1648 (2)	0.07161 (15)	0.42218 (6)	0.0330 (4)	
C12	0.0487 (2)	−0.02714 (15)	0.42656 (6)	0.0353 (4)	
C13	0.1101 (3)	−0.13927 (16)	0.43272 (7)	0.0421 (4)	
H13	0.2247	−0.1516	0.4335	0.050*	
C14	0.0055 (3)	−0.23210 (16)	0.43770 (8)	0.0476 (5)	
H14	0.0477	−0.3083	0.4422	0.057*	
C15	−0.1609 (3)	−0.21421 (18)	0.43615 (7)	0.0466 (5)	
C16	−0.2252 (3)	−0.10405 (19)	0.42947 (8)	0.0512 (5)	
H16	−0.3399	−0.0925	0.4280	0.061*	
C17	−0.1191 (3)	−0.01104 (17)	0.42499 (8)	0.0436 (4)	
H17	−0.1618	0.0651	0.4208	0.052*	
C18	−0.3900 (3)	−0.33373 (16)	0.41261 (7)	0.0440 (4)	
C19	−0.3950 (3)	−0.3008 (2)	0.36259 (8)	0.0556 (5)	
H19	−0.3112	−0.2534	0.3488	0.067*	
C20	−0.5230 (3)	−0.3373 (2)	0.33277 (9)	0.0665 (7)	
H20	−0.5275	−0.3144	0.2983	0.080*	
C21	−0.6440 (3)	−0.4065 (2)	0.35234 (11)	0.0697 (7)	
H21	−0.7308	−0.4327	0.3315	0.084*	
C22	−0.6383 (3)	−0.4377 (2)	0.40272 (10)	0.0657 (7)	
H22	−0.7229	−0.4843	0.4166	0.079*	
C23	−0.5117 (3)	−0.40221 (19)	0.43289 (8)	0.0536 (5)	
H23	−0.5079	−0.4245	0.4674	0.064*	
C24	0.0591 (3)	0.2062 (2)	0.55989 (7)	0.0537 (5)	
H24A	−0.0567	0.2248	0.5644	0.064*	
H24B	0.1247	0.2763	0.5652	0.064*	
H24C	0.0914	0.1466	0.5844	0.064*	
C25	0.0032 (3)	0.2714 (2)	0.20238 (8)	0.0599 (6)	
H25A	0.0191	0.2106	0.1770	0.072*	
H25B	0.0223	0.3477	0.1870	0.072*	
H25C	−0.1087	0.2677	0.2153	0.072*	
C26	0.1933 (2)	0.11466 (16)	0.31671 (6)	0.0340 (4)	
C27	0.3730 (2)	0.11156 (18)	0.30852 (6)	0.0375 (4)	
C28	0.4689 (2)	0.2093 (2)	0.31499 (7)	0.0458 (5)	
H28	0.4203	0.2820	0.3230	0.055*	
C29	0.6378 (3)	0.2002 (3)	0.30959 (9)	0.0597 (6)	
H29	0.7048	0.2666	0.3146	0.072*	
C30	0.7076 (3)	0.0954 (3)	0.29705 (9)	0.0633 (6)	
H30	0.8226	0.0898	0.2935	0.076*	
C31	0.6119 (3)	−0.0014 (2)	0.28961 (10)	0.0641 (6)	
H31	0.6606	−0.0733	0.2804	0.077*	
C32	0.4452 (3)	0.0062 (2)	0.29560 (9)	0.0519 (5)	
H32	0.3793	−0.0609	0.2909	0.062*	

### Atomic displacement parameters (Å^2^)

**Table:**

	*U*^11^	*U*^22^	*U*^33^	*U*^12^	*U*^13^	*U*^23^
O1	0.0357 (7)	0.0423 (7)	0.0407 (6)	0.0005 (6)	−0.0008 (5)	−0.0043 (5)
O2	0.0731 (11)	0.0508 (8)	0.0652 (9)	−0.0268 (8)	−0.0251 (9)	0.0206 (7)
O3	0.0606 (9)	0.0469 (7)	0.0309 (6)	0.0056 (7)	0.0013 (6)	−0.0039 (5)
O4	0.0563 (9)	0.0850 (11)	0.0335 (6)	0.0205 (9)	0.0023 (6)	0.0061 (7)
O5	0.0376 (7)	0.0425 (7)	0.0486 (7)	−0.0058 (6)	0.0001 (6)	−0.0108 (6)
C1	0.0326 (9)	0.0323 (8)	0.0353 (8)	−0.0049 (7)	0.0005 (7)	−0.0028 (7)
C2	0.0385 (9)	0.0365 (9)	0.0373 (8)	−0.0046 (8)	0.0015 (8)	−0.0042 (7)
C3	0.0465 (11)	0.0398 (9)	0.0423 (10)	−0.0036 (9)	0.0075 (9)	−0.0118 (8)
C4	0.0442 (10)	0.0293 (8)	0.0562 (11)	−0.0002 (8)	0.0080 (9)	−0.0082 (8)
C5	0.0358 (9)	0.0293 (8)	0.0492 (10)	−0.0041 (8)	0.0034 (8)	−0.0003 (7)
C6	0.0432 (10)	0.0326 (9)	0.0594 (11)	0.0010 (8)	0.0046 (9)	0.0058 (8)
C7	0.0460 (11)	0.0468 (10)	0.0497 (10)	0.0018 (10)	0.0007 (9)	0.0144 (9)
C8	0.0363 (10)	0.0498 (10)	0.0386 (9)	−0.0014 (9)	0.0035 (8)	0.0065 (8)
C9	0.0296 (8)	0.0373 (9)	0.0372 (9)	−0.0028 (8)	0.0005 (7)	0.0010 (7)
C10	0.0305 (8)	0.0301 (8)	0.0377 (8)	−0.0050 (7)	0.0012 (7)	−0.0025 (7)
C11	0.0395 (9)	0.0338 (8)	0.0257 (7)	−0.0016 (7)	−0.0016 (7)	−0.0032 (6)
C12	0.0418 (10)	0.0333 (8)	0.0308 (8)	−0.0028 (8)	−0.0026 (7)	−0.0009 (7)
C13	0.0450 (10)	0.0371 (9)	0.0441 (10)	0.0008 (9)	−0.0056 (9)	−0.0009 (8)
C14	0.0607 (13)	0.0321 (9)	0.0501 (10)	−0.0026 (9)	−0.0120 (10)	0.0028 (8)
C15	0.0568 (12)	0.0402 (10)	0.0429 (9)	−0.0141 (9)	−0.0099 (9)	0.0061 (8)
C16	0.0432 (11)	0.0484 (11)	0.0619 (12)	−0.0089 (10)	−0.0056 (10)	0.0059 (10)
C17	0.0440 (10)	0.0346 (9)	0.0523 (11)	−0.0014 (8)	−0.0006 (9)	0.0021 (8)
C18	0.0490 (11)	0.0372 (9)	0.0457 (10)	−0.0064 (9)	−0.0048 (9)	0.0007 (8)
C19	0.0554 (13)	0.0614 (13)	0.0500 (11)	−0.0127 (12)	−0.0002 (10)	0.0091 (10)
C20	0.0739 (17)	0.0763 (15)	0.0492 (12)	−0.0144 (15)	−0.0147 (12)	0.0059 (11)
C21	0.0610 (15)	0.0718 (15)	0.0764 (15)	−0.0165 (14)	−0.0230 (13)	0.0024 (13)
C22	0.0521 (13)	0.0650 (15)	0.0799 (16)	−0.0196 (13)	−0.0008 (12)	0.0079 (12)
C23	0.0609 (13)	0.0502 (11)	0.0498 (11)	−0.0139 (11)	0.0020 (10)	0.0055 (9)
C24	0.0649 (14)	0.0632 (12)	0.0329 (9)	0.0060 (12)	0.0038 (9)	−0.0084 (9)
C25	0.0696 (15)	0.0599 (13)	0.0503 (11)	0.0168 (13)	−0.0132 (11)	−0.0093 (10)
C26	0.0340 (9)	0.0408 (9)	0.0273 (7)	−0.0001 (8)	−0.0015 (7)	−0.0035 (7)
C27	0.0330 (9)	0.0494 (10)	0.0301 (8)	0.0016 (8)	−0.0010 (7)	0.0007 (8)
C28	0.0383 (10)	0.0595 (12)	0.0395 (9)	−0.0057 (10)	0.0019 (8)	−0.0073 (9)
C29	0.0379 (11)	0.0877 (17)	0.0536 (12)	−0.0151 (12)	0.0018 (9)	−0.0076 (12)
C30	0.0320 (10)	0.0962 (19)	0.0618 (13)	0.0077 (13)	0.0013 (10)	0.0102 (14)
C31	0.0475 (12)	0.0696 (15)	0.0753 (15)	0.0188 (12)	0.0093 (12)	0.0099 (13)
C32	0.0427 (11)	0.0504 (12)	0.0625 (13)	0.0074 (10)	0.0058 (10)	0.0041 (10)

### Geometric parameters (Å, º)

**Table:**

O1—C11	1.220 (2)	C16—C17	1.384 (3)
O2—C15	1.387 (2)	C16—H16	0.9500
O2—C18	1.391 (2)	C17—H17	0.9500
O3—C2	1.365 (2)	C18—C19	1.379 (3)
O3—C24	1.429 (2)	C18—C23	1.380 (3)
O4—C8	1.372 (2)	C19—C20	1.378 (3)
O4—C25	1.406 (3)	C19—H19	0.9500
O5—C26	1.209 (2)	C20—C21	1.374 (4)
C1—C2	1.381 (2)	C20—H20	0.9500
C1—C10	1.429 (2)	C21—C22	1.383 (4)
C1—C11	1.518 (2)	C21—H21	0.9500
C2—C3	1.412 (3)	C22—C23	1.372 (3)
C3—C4	1.359 (3)	C22—H22	0.9500
C3—H3	0.9500	C23—H23	0.9500
C4—C5	1.414 (3)	C24—H24A	0.9800
C4—H4	0.9500	C24—H24B	0.9800
C5—C6	1.413 (3)	C24—H24C	0.9800
C5—C10	1.428 (2)	C25—H25A	0.9800
C6—C7	1.368 (3)	C25—H25B	0.9800
C6—H6	0.9500	C25—H25C	0.9800
C7—C8	1.401 (3)	C26—C27	1.489 (2)
C7—H7	0.9500	C27—C28	1.383 (3)
C8—C9	1.379 (3)	C27—C32	1.392 (3)
C9—C10	1.427 (2)	C28—C29	1.396 (3)
C9—C26	1.508 (3)	C28—H28	0.9500
C11—C12	1.487 (2)	C29—C30	1.375 (4)
C12—C17	1.388 (3)	C29—H29	0.9500
C12—C13	1.394 (3)	C30—C31	1.377 (4)
C13—C14	1.376 (3)	C30—H30	0.9500
C13—H13	0.9500	C31—C32	1.378 (3)
C14—C15	1.380 (3)	C31—H31	0.9500
C14—H14	0.9500	C32—H32	0.9500
C15—C16	1.384 (3)		
			
C15—O2—C18	120.31 (15)	C12—C17—H17	119.4
C2—O3—C24	118.06 (16)	C19—C18—C23	120.6 (2)
C8—O4—C25	117.51 (17)	C19—C18—O2	123.43 (19)
C2—C1—C10	120.00 (15)	C23—C18—O2	115.69 (18)
C2—C1—C11	116.07 (15)	C20—C19—C18	119.3 (2)
C10—C1—C11	123.92 (15)	C20—C19—H19	120.3
O3—C2—C1	115.28 (15)	C18—C19—H19	120.3
O3—C2—C3	123.22 (16)	C21—C20—C19	120.7 (2)
C1—C2—C3	121.50 (16)	C21—C20—H20	119.7
C4—C3—C2	119.07 (17)	C19—C20—H20	119.7
C4—C3—H3	120.5	C20—C21—C22	119.4 (2)
C2—C3—H3	120.5	C20—C21—H21	120.3
C3—C4—C5	121.89 (17)	C22—C21—H21	120.3
C3—C4—H4	119.1	C23—C22—C21	120.7 (2)
C5—C4—H4	119.1	C23—C22—H22	119.6
C6—C5—C4	121.11 (17)	C21—C22—H22	119.6
C6—C5—C10	119.57 (16)	C22—C23—C18	119.3 (2)
C4—C5—C10	119.32 (16)	C22—C23—H23	120.3
C7—C6—C5	121.75 (17)	C18—C23—H23	120.3
C7—C6—H6	119.1	O3—C24—H24A	109.5
C5—C6—H6	119.1	O3—C24—H24B	109.5
C6—C7—C8	118.78 (17)	H24A—C24—H24B	109.5
C6—C7—H7	120.6	O3—C24—H24C	109.5
C8—C7—H7	120.6	H24A—C24—H24C	109.5
O4—C8—C9	115.46 (17)	H24B—C24—H24C	109.5
O4—C8—C7	122.59 (17)	O4—C25—H25A	109.5
C9—C8—C7	121.95 (17)	O4—C25—H25B	109.5
C8—C9—C10	120.18 (17)	H25A—C25—H25B	109.5
C8—C9—C26	115.71 (16)	O4—C25—H25C	109.5
C10—C9—C26	123.98 (15)	H25A—C25—H25C	109.5
C9—C10—C5	117.68 (15)	H25B—C25—H25C	109.5
C9—C10—C1	124.26 (15)	O5—C26—C27	121.40 (18)
C5—C10—C1	118.03 (15)	O5—C26—C9	119.47 (15)
O1—C11—C12	121.49 (16)	C27—C26—C9	119.13 (17)
O1—C11—C1	120.45 (16)	C28—C27—C32	119.81 (18)
C12—C11—C1	118.04 (15)	C28—C27—C26	121.65 (19)
C17—C12—C13	118.98 (18)	C32—C27—C26	118.52 (19)
C17—C12—C11	122.00 (17)	C27—C28—C29	119.3 (2)
C13—C12—C11	119.02 (17)	C27—C28—H28	120.3
C14—C13—C12	120.31 (19)	C29—C28—H28	120.3
C14—C13—H13	119.8	C30—C29—C28	120.2 (2)
C12—C13—H13	119.8	C30—C29—H29	119.9
C13—C14—C15	119.80 (19)	C28—C29—H29	119.9
C13—C14—H14	120.1	C29—C30—C31	120.5 (2)
C15—C14—H14	120.1	C29—C30—H30	119.8
C14—C15—C16	121.14 (19)	C31—C30—H30	119.8
C14—C15—O2	116.44 (19)	C30—C31—C32	119.8 (2)
C16—C15—O2	122.33 (19)	C30—C31—H31	120.1
C17—C16—C15	118.6 (2)	C32—C31—H31	120.1
C17—C16—H16	120.7	C31—C32—C27	120.3 (2)
C15—C16—H16	120.7	C31—C32—H32	119.8
C16—C17—C12	121.12 (19)	C27—C32—H32	119.8
C16—C17—H17	119.4		
			
C24—O3—C2—C1	174.60 (18)	O1—C11—C12—C13	7.3 (3)
C24—O3—C2—C3	−5.9 (3)	C1—C11—C12—C13	−171.04 (16)
C10—C1—C2—O3	−179.94 (15)	C17—C12—C13—C14	−0.7 (3)
C11—C1—C2—O3	1.2 (2)	C11—C12—C13—C14	178.93 (16)
C10—C1—C2—C3	0.6 (3)	C12—C13—C14—C15	0.6 (3)
C11—C1—C2—C3	−178.30 (16)	C13—C14—C15—C16	0.2 (3)
O3—C2—C3—C4	−176.71 (18)	C13—C14—C15—O2	−176.52 (16)
C1—C2—C3—C4	2.7 (3)	C18—O2—C15—C14	−133.4 (2)
C2—C3—C4—C5	−2.6 (3)	C18—O2—C15—C16	49.9 (3)
C3—C4—C5—C6	178.59 (19)	C14—C15—C16—C17	−0.8 (3)
C3—C4—C5—C10	−0.8 (3)	O2—C15—C16—C17	175.70 (19)
C4—C5—C6—C7	179.8 (2)	C15—C16—C17—C12	0.7 (3)
C10—C5—C6—C7	−0.8 (3)	C13—C12—C17—C16	0.1 (3)
C5—C6—C7—C8	−0.8 (3)	C11—C12—C17—C16	−179.56 (17)
C25—O4—C8—C9	141.6 (2)	C15—O2—C18—C19	29.0 (3)
C25—O4—C8—C7	−38.4 (3)	C15—O2—C18—C23	−157.0 (2)
C6—C7—C8—O4	−179.69 (19)	C23—C18—C19—C20	−0.3 (4)
C6—C7—C8—C9	0.3 (3)	O2—C18—C19—C20	173.4 (2)
O4—C8—C9—C10	−178.22 (17)	C18—C19—C20—C21	−0.5 (4)
C7—C8—C9—C10	1.8 (3)	C19—C20—C21—C22	1.2 (4)
O4—C8—C9—C26	−2.1 (2)	C20—C21—C22—C23	−1.3 (4)
C7—C8—C9—C26	177.96 (17)	C21—C22—C23—C18	0.6 (4)
C8—C9—C10—C5	−3.3 (3)	C19—C18—C23—C22	0.2 (4)
C26—C9—C10—C5	−179.09 (16)	O2—C18—C23—C22	−174.0 (2)
C8—C9—C10—C1	174.74 (17)	C8—C9—C26—O5	−94.6 (2)
C26—C9—C10—C1	−1.0 (3)	C10—C9—C26—O5	81.4 (2)
C6—C5—C10—C9	2.8 (3)	C8—C9—C26—C27	85.2 (2)
C4—C5—C10—C9	−177.78 (17)	C10—C9—C26—C27	−98.9 (2)
C6—C5—C10—C1	−175.38 (17)	O5—C26—C27—C28	−176.63 (17)
C4—C5—C10—C1	4.1 (2)	C9—C26—C27—C28	3.6 (3)
C2—C1—C10—C9	178.04 (17)	O5—C26—C27—C32	1.3 (3)
C11—C1—C10—C9	−3.2 (3)	C9—C26—C27—C32	−178.47 (17)
C2—C1—C10—C5	−3.9 (2)	C32—C27—C28—C29	−1.5 (3)
C11—C1—C10—C5	174.87 (15)	C26—C27—C28—C29	176.44 (18)
C2—C1—C11—O1	−100.0 (2)	C27—C28—C29—C30	1.2 (3)
C10—C1—C11—O1	81.2 (2)	C28—C29—C30—C31	0.1 (4)
C2—C1—C11—C12	78.3 (2)	C29—C30—C31—C32	−1.1 (4)
C10—C1—C11—C12	−100.50 (19)	C30—C31—C32—C27	0.8 (4)
O1—C11—C12—C17	−173.09 (18)	C28—C27—C32—C31	0.5 (3)
C1—C11—C12—C17	8.6 (2)	C26—C27—C32—C31	−177.5 (2)

### Hydrogen-bond geometry (Å, º)

Cg1, Cg2 and Cg3 are the centroids of the C27–C32, C12–C17 and C5–C10 rings, respectively.

**Table:**

*D*—H···*A*	*D*—H	H···*A*	*D*···*A*	*D*—H···*A*
C30—H30···O5^i^	0.95	2.52	3.423 (3)	158
C7—H7···O5^ii^	0.95	2.37	3.304 (3)	168
C25—H25*B*···O5^ii^	0.98	2.34	3.122 (3)	136
C3—H3···O1^iii^	0.95	2.40	3.310 (2)	160
C20—H20···*Cg*1^iv^	0.95	2.84	3.652 (3)	144
C23—H23···*Cg*2^v^	0.95	2.76	3.628 (2)	151
C29—H29···*Cg*3^i^	0.95	2.85	3.652 (3)	142

Symmetry codes: (i) *x*+1, *y*, *z*; (ii) −*x*, *y*+1/2, −*z*+1/2; (iii) *x*−1/2, −*y*+1/2, −*z*+1; (iv) −*x*, *y*−1/2, −*z*+1/2; (v) *x*−1/2, −*y*−1/2, −*z*+1.
